# Ru/Al Multilayers Integrate Maximum Energy Density and Ductility for Reactive Materials

**DOI:** 10.1038/srep19535

**Published:** 2016-01-29

**Authors:** K. Woll, A. Bergamaschi, K. Avchachov, F. Djurabekova, S. Gier, C. Pauly, P. Leibenguth, C. Wagner, K. Nordlund, F. Mücklich

**Affiliations:** 1 Functional Materials, Department of Materials Science, Saarland University, 66123 Saarbrücken, Germany; 2 Paul Scherrer Institute, 5232 Villigen-PSI, Switzerland; 3 Materials Physics, Department of Physics, University of Helsinki, 00014 Helsinki, Finland; 4 Experimental Physics, Saarland University, 66123 Saarbrücken, Germany

## Abstract

Established and already commercialized energetic materials, such as those based on Ni/Al for joining, lack the adequate combination of high energy density and ductile reaction products. To join components, this combination is required for mechanically reliable bonds. In addition to the improvement of existing technologies, expansion into new fields of application can also be anticipated which triggers the search for improved materials. Here, we present a comprehensive characterization of the key parameters that enables us to classify the Ru/Al system as new reactive material among other energetic systems. We finally found that Ru/Al exhibits the unusual integration of high energy density and ductility. For example, we measured reaction front velocities up to 10.9 (±0.33) ms^−1^ and peak reaction temperatures of about 2000 °C indicating the elevated energy density. To our knowledge, such high temperatures have never been reported in experiments for metallic multilayers. *In situ* experiments show the synthesis of a single-phase B2-RuAl microstructure ensuring improved ductility. Molecular dynamics simulations corroborate the transformation behavior to RuAl. This study fundamentally characterizes a Ru/Al system and demonstrates its enhanced properties fulfilling the identification requirements of a novel nanoscaled energetic material.

Nanometric reactive multilayers are capable of long-term chemical energy storage. The energy is released after local ignition during rapid self-propagating reactions (see reviews^1^,^2^,^3^). The reactions form intermetallic compounds within a micron-sized reaction front traveling along the multilayer (see Fig. 1(a)). This type of energy release makes reactive multilayers attractive as localized heat sources. Reactive multilayers of various chemistries have been explored to adjust the reaction properties to the requirements given by the application^4^,^5^,^6^,^7^,^8^,^9^,^10^,^11^,^12^,^13^. For example, in the NanoBond^TM^ process, Ni/Al foil enables bonding of components at the smallest scale by building self-forming joints where only the joint interface exhibits heat-up. The need for external heat sources is eliminated, paving the way for accelerated production, e.g. in microelectromechanical systems (MEMS)^13^. However, the development of new joining strategies or the exploitation of novel applications requires three conflicting properties of the energetic systems^14^: 1.) reactions with maximal energy density (ED) leading to high reaction temperatures *T*_*f*_, 2.) reactions to single-phase microstructures, and 3.) reaction products with room temperature (RT) ductility.

Currently, no system adequately combines these properties. The analysis of the most widely used systems Ni/Al and Pd/Al (e.g. for soldering) illustrates the conflict. Bonding at the micron scale needs high ED to produce stable reaction progress and high *T*_*f*_. In this respect, equiatomic systems with maximal ED, such as Pd/Al (10.8 kJcm^−3^ 15) are preferred. Theoretical calculations show that T_f_s are around 2,800 °C^15^. However, there is no experimental evidence. When reacted as freestanding multilayers those foils lose mechanical integrity. The reasons for the loss in material integrity are currently an open research topic. Potential scenarios include processes like metal evaporation during the reaction, full melting due to the high heat of formation, and the formation of volatile precious metal oxides. The example reveals the drawback of too high EDs and *T*_*f*_. However, motivated by the abovementioned joining or MEMS applications, reactions of optimal energetic materials should reach temperatures close to the maximal temperature of Pd/Al and simultaneously preserve foil integrity.

High temperatures also cause composition variation during reaction. In this sense, equiatomic Ni/Al is a better choice than Pd/Al. The evolving B2-NiAl intermetallic is also stable for off-stoichiometric^16^,^17^ compositions guaranteeing robust fabrication of single-phase microstructures. By contrast, systems like Pd/Al or Pt/Al produce complex equilibrium or metastable phases around the equiatomic composition^8^,^18^,^19^. Destabilization of the reacted microstructures may occur. Consequently, optimal reactive materials should tolerate stoichiometric variations while forming the equilibrium compound.

Whereas Ni/Al and Pd/Al each show advantages for one of the aforementioned key properties, they exhibit no RT-product-ductility. Thus, the systems are inefficient for applications requiring mechanical reliability. It is generally known that intermetallics are highly brittle^16^,^20^ eventually narrowing down the material selection substantially. However, there is a strong demand for systems producing RT-ductile intermetallics^14^. Their utilization would enable a technology expansion into fields requiring mechanical reliability at RT. In fact, researchers discovered a family of ductile B2 compounds^20^. However, self-propagating reactions of those systems produce multiphase microstructures because these phases are line compounds exhibiting no off-stoichiometry tolerance^12^. Furthermore, they have the lowest EDs (<6.6 kJcm^−3^). Hence, we are left to conclude: to date no energetic material combines high energy density with maximal ductility.

Since Ni/Al is currently the only commercially available and already used metallic energetic material, we took its properties as a benchmark, theoretically explored various binary metallic systems, and identified the equiatomic Ru/Al system to show the best combination of aforementioned properties (see our reviews^21^,^22^). First, it combines high ED (8.0 kJcm^−3^) with the formation of the intermetallic RuAl also for off-stoichiometric compositions. Second, we calculated the adiabatic temperature T_ad_ to infer the maximal possible T_f_. T_ad_ = 1,977 °C which is 339 °C higher than that of Ni/Al (1,638 °C^1^) enabling us to anticipate substantially higher T_f_s for Ru/Al. Third, polycrystalline RuAl exhibits exceptional RT-ductility^22^. This is unusual for intermetallics (see Fig. 1(b)). We plot the mechanical anisotropy against Poisson’s ratio^20^. Ductile B2 compounds cluster in the indicated range (circular dotted line in Fig. 1(b)) also including RuAl leaving out brittle phases like NiAl, PtAl, and PdAl. The comparison of the EDs (data point diameter of every intermetallic in Fig. 1(b)) reveals the maximal value for the Ru/Al system among the ductile phases. We therefore hypothesize: Ru/Al is the system with maximal ED and maximal ductility.

## Results and Discussion

Guided by our hypothesis, we determine and analyze the key properties of reactive Ru/Al multilayer foils. The analysis enables us to relate the Ru/Al system to the already known reactive systems mentioned above. For this purpose, we follow a twofold approach. On the one hand we measure well accepted standard parameters such as front velocities or reaction temperatures. The parameters allow us to classify Ru/Al as reactive material in general and in particular in terms of energy density. Higher reaction velocities and temperatures compared to Ni/Al provide experimental evidence for the higher energy density. On the other hand we utilize a combined approach of modern *in situ* techniques, microstructural analysis and atomistic simulations to explore the reaction path and the final microstructure. We found a single-step reaction path and a single-phase as-reacted microstructure which we interpret as essential indicators for improved ductility.

### Measures for Energy density

**Figure 2:**
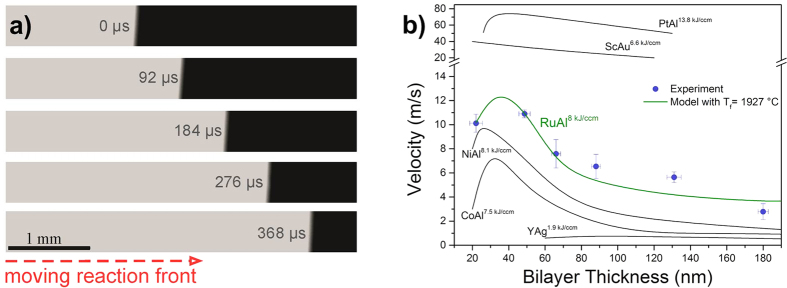
Measured velocities provide evidence for the high energy density stored in Ru/Al multilayers. (**a**) Successive still images of a reaction front which moves from the left to the right (Λ = 88 nm). Time intervals are relative to the first image. (**b)** Measured (blue points) and modeled (full green line) reaction velocity as a function of Λ. Modeling using Equation (1) was performed with ω = 5 nm, E = 205 kJ/mol, λ = 1.5·10^−5^ m^2^/s, A = 0.15, T_ad_ = 1977 °C and T_f_ = 1927 °C. A comparison of the reaction velocity in the Ru/Al system with literature data of Y/Ag ^21^, Sc/Au ^21^ Co/Al ^7^, Ni/Al ^5^ and Pt/Al ^8^ is also shown. Consistent with the similar stored energy, the velocities of Ru/Al are close to the values of Ni/Al.

Generally, the velocity and the maximal temperature of a self-propagating reaction are indicators for the energy density. In a first step we, thus, measure the front velocity for the Ru/Al multilayers. Figure 2(a) depicts successive high-speed camera images of a sample with bilayer thickness Λ = 88 nm. The reaction, characterized by the bright contrast, moves from left to right. Figure 2(b) shows the measured velocity as a function of bilayer thickness Λ. The reaction velocity increases from 2.8 to 10.9 ms^−1^ with decreasing Λ. At Λ ≈ 22 nm, the velocity drops down to 10.1 ms^−1^. This behavior is a common observation for self-propagating reactions in metallic multilayers as the following two mechanisms prevail^6^. As the reaction propagates, atomic diffusion normal to the individual layers occurs. With decreasing Λ the diffusion distances also decrease which result in faster reaction rates, i.e. velocities. However, during multilayer fabrication, chemical energy is lost due to interdiffusion across the interfaces resulting in velocity reduction. For Ru/Al multilayers, the interdiffusion length is reported to be 0.8 up to 5 nm^23^. For a decrease in Λ the premixing effect becomes more pronounced and contributes to the velocity. Due to this balance, a critical Λ exists where premixing slows the reaction rate. Mann *et al.* developed an analytical model which considers these aspects^6^. Accordingly, the velocity v can be modeled as a function of Λ using Equation (1)


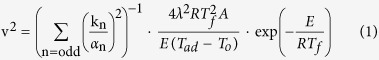


where k_n_ and α_n_ are the Fourier coefficients and the eigenvalues of the Fourier series of the multilayer composition profile. λ, T_f_, A, E, T_ad_, T_0_ and R are the thermal diffusion coefficient, the reaction temperature, an Arrhenius prefactor, the activation energy for atomic diffusion, the adiabatic reaction temperature, the preheating temperature and the gas constant, respectively. Using Eq. (1) to model the velocity data, an average reaction temperature of 1,927 °C can be estimated (the used parameters are given in the figure caption). Figure 2(b) also compares the velocities to literature data of other important systems. An increasing amount of stored chemical energy results in higher front velocities. This trend is generally observed in reactive systems^12^. Therefore, the burning rates of Ru/Al multilayers consistently range between those of Ni/Al and Pt/Al and the velocity measurements are a first experimental evidence of the higher energy density of the system compared with the already commercially distributed Ni/Al.

**Figure 3:**
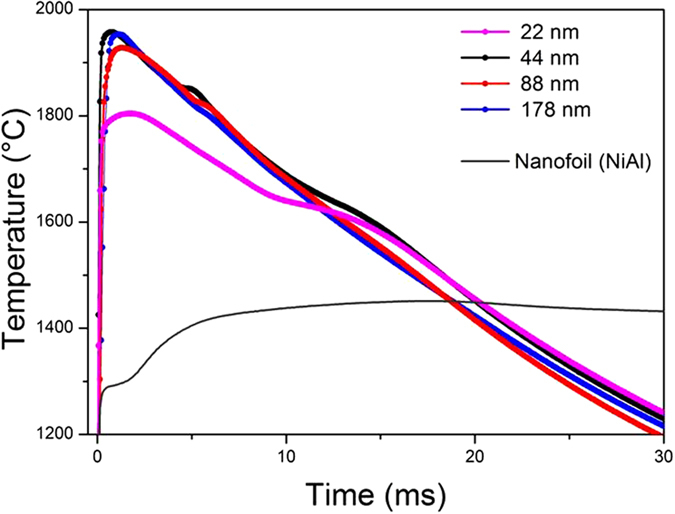
Ru/Al multilayers react under substantially higher temperatures than Ni/Al. Temperature profiles during reaction in air for different Λ. Depending on Λ the peak temperature varies between 1800 and 1950 °C. Note the temperature increase of at least 450 °C when compared to the measured reaction temperatures in a commercial Ni/Al foil (grey line).

For the next step of energy density validation, we measure the reaction temperature and captured temperature profiles during the reaction in air using high-speed pyrometry, Fig. 3. We precisely discuss the temperature profiles in Supplementary 1. Here we only report the most important characteristics. The measurements reveal peak temperatures between 1,800 °C and 1,950 °C depending on Λ. The result is in reasonable agreement with the assessment based on the velocity data. Considering the adiabatic temperature of 1,977 °C, which denotes the theoretically maximal possible temperature, we generally conclude that the reaction occurs under nearly adiabatic conditions. Our foils thus react under maximal possible temperatures. In greater detail, we observe a temperature drop for the small-Λ multilayers. The loss of chemical energy due to fabrication-induced interface interdiffusion causes the latter drop. However, these details aside, the substantially higher temperatures compared to that of Ni/Al are most intriguing because Ru/Al foils store the same chemical ED of ~8 kJ/cm^−3^. Here, the Ru/Al system notably benefits from the exceptionally high melting temperature T_m_ of the RuAl intermetallic (2,033 °C^21^). Whereas T_ad_ for the Ni/Al system equals T_m_ of the intermtallic NiAl (1,638 °C^17^), T_ad_ for the Ru/Al system is slightly lower than T_m_ of RuAl. In the former case, the stored energy enables foil heat-up only to the product melting point where product melting occurs. This detrimentally consumes a part of the energy and impedes further heat-up. Conversely for Ru/Al, the lower T_ad_ does not cause compound melting. Hence T_f_ is not limited enabling the Ru/Al foils to reach significantly higher peak temperatures.

### Indicators for Ductility

Polycrystalline RuAl with a homogeneous microstructure of equiaxed grains exhibits unusual high ductility^24^,^25^,^26^. Hence, we define two indicators mandatory for ductility. As a first indicator, we consider a one-step reaction pathway to RuAl. The absence of other intermetallics than RuAl during the reaction is important to develop a homogeneous microstructure with equiaxed grains. Transient secondary phases would have to turn to RuAl in subsequent reaction stages, e.g. via peritectic transformations resulting in non-homogeneous microstructures of the reacted foil. Thus, we explore the reaction pathway which should directly lead to RuAl. As a second indicator, we define a single phase RuAl microstructure of the reacted foil. This is mandatory because the presence of any second Ru/Al intermetallic would substantially reduce the overall ductility.

**Figure 4:**
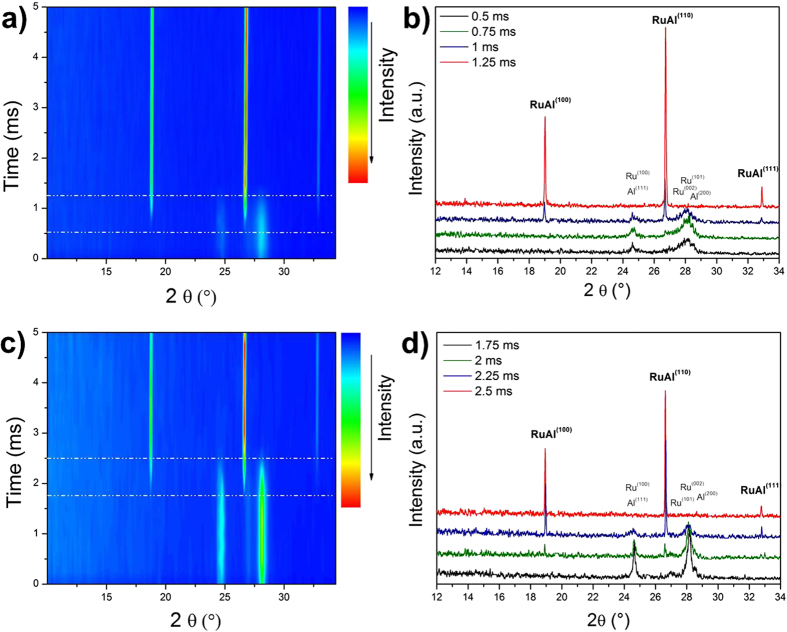
High-speed X-ray diffraction analysis of reaction progression in Ru/Al multilayer indicates improved ductility. (**a**) Time development of the diffracted intensity for a multilayer with Λ = 22 nm. A phase transformation occurs between 0.5 and 1.25 ms after ignition. (**b**) Successive diffractograms of the reaction zone in a). During transformation, only reflections of Al, Ru and RuAl are detectable. This indicates the direct transformation of the elements to RuAl. (**c**) Time progression of the diffracted intensity for a multilayer sample with Λ = 178 nm. The phase transformation now occurs between 1.75 and 2.5 ms after ignition which is consistent with the decreased reaction velocity. (**d**) Successive diffractograms during the transformation indicated in **c**). Again, only the peaks of Al, Ru and RuAl are observable. Note the significant peak broadening of the elemental reflections during reaction.

First we explore the reaction pathway to the final RuAl phase which is, for the moment, unknown. For the fast rates of self-propagating reactions the phase sequence is unseizable by means of slow heating experiments or *ex situ* methods^27^ as the high front velocities imply high reaction rates and constrained conditions for nucleation and growth. Hence, advanced *in situ* techniques with high temporal and/or spatial resolution need to be applied. To take on this issue in reactive multilayers only little work has been done so far. Latest studies on reactive Ni/Al foils used dynamic transmission electron microscopy (DTEM)^28^,^29^ or synchrotron radiation^27^,^30^. We performed *in situ* time-resolved synchrotron experiments in air using a high-speed MYTHEN II micro strip detector^31^. In contrast to the mentioned *in situ* studies, this detector allows a continuous analysis of the temporal phase evolution. Figure 4(a) shows the diffracted intensity over time in a Ru/Al multilayer with Λ = 22 nm. Only reflections of the elements are observed during the first 0.75 ms. Between 0.75 ms and 1.25 ms the reaction front approaches the focus point of the X-Ray beam and new reflections occur. Simultaneously, the elemental peaks decrease. This indicates a phase transformation where the elements react to a final phase. Between 1.25 ms and 5 ms, no new peaks are visible. Phase identification during the transformation period (0.75–1.25 ms) is done in Fig. 4(b). At 0.5 ms after ignition only the strong overlapping Ru{100} and Al{111} peaks at diffraction angles of 24.64° and the overlapping Ru{002}, Ru{101} and Al{200} peaks between 27 and 28.5° are visible. RuAl reflections at 19, 26.7 and 32.8° are clearly observable for times >0.75 ms. We note, that the other binary Ru/Al compounds also have the main reflections in the plotted 2θ range. However, no intermetallic other than RuAl can be detected during the first 5 ms. Due to the detection limit of XRD it cannot be definitively ruled out that there are other crystalline phases present during the transformation in very small amounts (or for very short times). However, kinetic and thermodynamic considerations corroborate RuAl to be the only phase to form. First, we consider the atoms per unit cell of the individual Ru/Al compounds to deduce a kinetic argument. A higher number of atoms per unit cell or more complex crystal structures increase nucleation times and act as a kinetic barrier for nucleation. Since diffusion and nucleation times in self-propagating reactions are short, phases with the least atoms per unit cell and simple crystal structure are preferred. RuAl with its cubic structure and two atoms per cell is therefore strongly promoted over other possible Ru/Al compounds with more complex crystal structures. The latter have ten to 102 atoms per unit cell^32^. Second, the analysis of the thermal-to-mass-diffusion ratio also supports RuAl to be the only phase to form. The ratio of thermal to mass diffusion reaches values >10^3^ at *T*_*f*_ during initial heat-up^33^. Thermal transport along the foil purportedly dominates over mass diffusion perpendicular to the interfaces in the unreacted volume ahead of the reaction front. Substantial sublayer intermixing and compositional changes during initial heat-up can be neglected. Our MD simulations support this (see below). Ru-dissolution then only occurs close to or at *T*_*f*_ = 1950 °C where Al is liquid. Under these conditions Al dissolves up to 30 at.% Ru^21^. Once the solubility limit is reached, RuAl directly precipitates without preceding phase transformations. Third, we consider the driving force for nucleation to get a thermodynamic argument for the preferred RuAl formation. The driving force is proportional to the undercooling. T_m_ of all Ru/Al compounds except that of RuAl is exceeded that only creates a driving force for the RuAl nucleation. Fourth, the stability range of RuAl points to preferred nucleation^27^,^34^. Consequently, our XRD experiments strongly indicate the direct RuAl formation from the elements which was previously defined as a mandatory indicator for a ductile foil microstructure. Thus, the reaction fulfills the prerequisite to transform to a ductile foil.

The discussion of the reaction pathway considered so far the case of a bilayer thickness Λ of 22 nm. We answer now the question whether the reaction pathway changes with Λ. In this context we note, that the results of the first section demonstrate that a Λ variation enables us to tailor front velocity and temperature. Utilizing the latter effects we can adjust the reaction in accordance with the requirements of the application. However, changing Λ should not alter the reaction pathway of the self-propagating reactions. In fact, the pathway should be insensitive to Λ variation to still guarantee the absence of second phases.

In terms of the phase sequence, it is known from slow-heating experiments that Λ substantially affects the reaction pathway, e.g. for Ni/Al multilayers^35^,^36^. For the case of the fast-heating, self-propagating reaction we also performed *in situ* diffraction measurements on large-Λ samples (Λ = 178 nm). Figure 4(c) presents the results.

The reaction front does not approach the focus point of the X-Ray beam until 1.75 ms (compare 0.75 ms for the small Λ case in Fig. 4(a)). The increase in time is in accordance with the substantially decreased front velocity in the multilayers by a factor of ≈3 (compare Fig. 2). Then, the diffracted intensity changes and indicates the transformation of the foil. The transformation behavior is comparable with that of the small-Λ samples presented above. The results show no indication for another intermetallic formation except that of RuAl. Hence, the transformation behavior is indeed Λ-independent for Λ ≤ 178 nm.

To gain refined understanding of the RuAl formation, we used the individual diffractograms between 1.75 ms and 2.5 ms (see Fig. 4(d)) and qualitatively analyzed the temporal evolution of the peak width of the Ru{101} reflection at 28.17°. The peak width increases during the phase transformation. The broadening points to microstructural changes of the Ru-sublayers probably induced by the interfacial intermixing. Microstrain and grain size effects contribute to the temporal development of the peaks^37^,^38^. However, due to strong peak overlapping and the time-integrating characteristic of the individual diffractograms, quantification using profile fitting routines are generally misleading.

**Figure 5:**
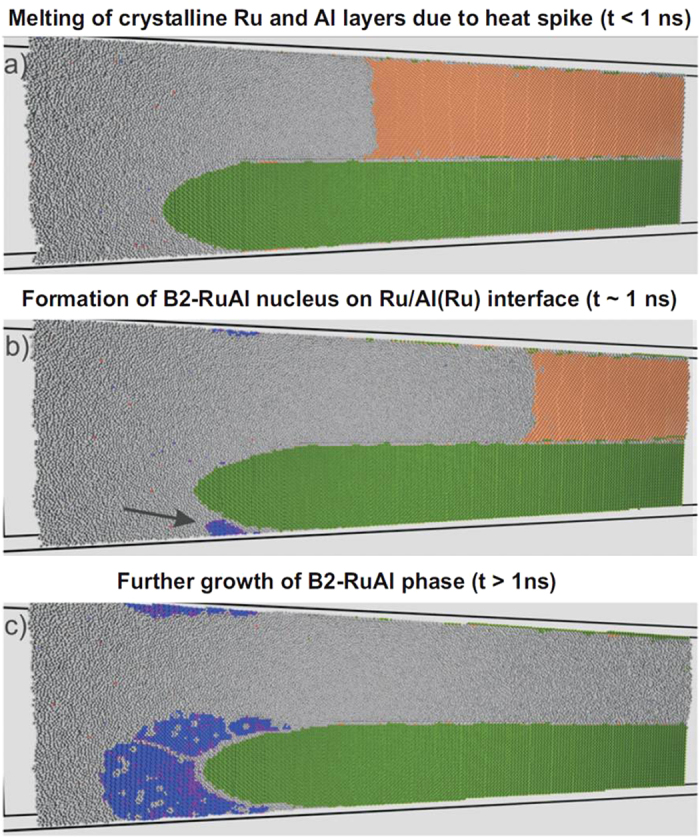
Molecular dynamics simulations of intermixing within Ru/Al multilayers corroborate the transformation behavior to RuAl required for improved ductility. Atoms are colored based on common neighbor analysis: coral atoms have FCC environment; green atoms HCP; blue atoms B2-BCC; purple atoms random-BCC and grey ones are not identified in structure (liquid). (**a**) The sample after a heat impulse was induced: left part of Ru/Al layers is melted, and then due to heat diffusion, melting of the Al layer happens. (**b**) A B2-RuAl nucleus forms on the solid Ru - liquid Ru-Al interface. There are few atoms in disordered BCC lattice on the periphery of the nucleus. (**c**) The growth of the B2-BCC RuAl phase starts from the formed nucleus. We observe quite uniform B2-BCC structure with several inclusions of vacancies (grey spots) and disordered BCC on periphery (purple spots). Full animated MD simulation is attached in the Supplementary.

Thus, we complementary chose the molecular dynamics (MD) simulation method to reveal the processes. The MD simulation also acts as a stand-alone tool to examine the transformations under the non-equilibrium conditions of the reaction. Simulating the first 2 ns after ignition, e.g. via an electrical spark, we identified three transformation stages (see Fig. 5 (a–c)). A full video is presented in the Supplementary Material. Here, we describe the three stages of transformation. Stage one describes ignition. Crystalline aluminum and ruthenium start melting locally (Fig. 5(a)). A liquid/solid front spreads rapidly in both layers. Unlike the Ru layer, the Al layer melts entirely within 1.7 ns. At this time, we stopped the simulation because the melting front cannot propagate further. Stage two comprises the onset of local RuAl-nucleation (Fig. 5(b)) within the ignition affected area. The RuAl nucleus has an ordered B2 lattice structure with some lattice defects predominantly on the nucleus periphery. The similarity of the close packed (110) B2 plane and the close packed (0002) HCP plane promotes RuAl nucleation between solid Ru and the liquid Al(Ru). In stage three the RuAl fraction increases by growth of the existing nucleus and the formation of new nuclei (Fig. 5(c)). The separation of different nuclei is seen on Fig. 5(c) at the grain boundaries. The formed B2 phase is homogeneous and contains some defects such as vacancies.

The MD simulations demonstrated simultaneous Ru dissolution into liquid Al and RuAl nucleation at the Ru/Al interface. Literature suggests a similar nucleation mechanism for NiAl in Ni/Al multilayers^27^. Against this background, we consider the peak broadening observed in our *in situ* experiments. The dissolution mechanism suggests that the Ru sublayers 1) strain (due to the 7% volume expansion of the surrounding Al) and 2) shrink due to dissolution. Both contributions probably lead to the peak broadening. Consequently, our MD simulations and *in-situ* experiments coincide in terms of the inferred mechanism.

The diffractograms of the *in situ* XRD study have also been used to estimate the reaction temperature as the peak positions correlate with the temperature. The RuAl{211} peak was chosen (being isolated and relatively high in angle). The final XRD patterns obtained at room temperature served as reference to calibrate the 2θ zero shift. The maximal error due to macrostrains is determined to be 72 °C under the assumption of a biaxial stress state. Using thermal expansion data of RuAl (α = 8.2 · 10^−6^ K^−1^)^39^ the maximal temperature during the reaction was calculated to be 1,980 °C. Hence, the velocity and the diffraction experiments are consistent with the measured temperatures of maximal 1950 °C.

**Figure 6:**
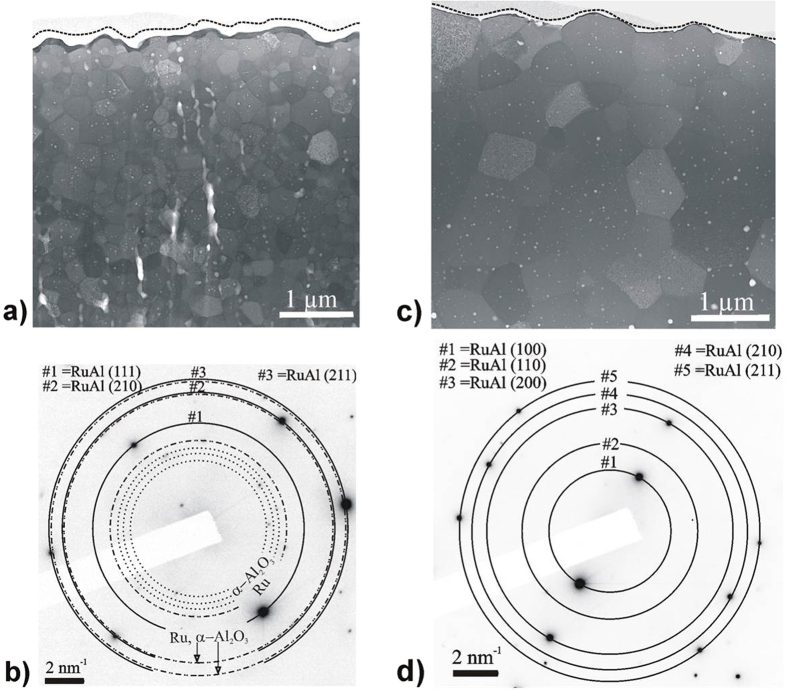
Evidence of single phase, as-reacted foil microstructures as requirement for improved ductility. (**a**,**c**) STEM cross-sectional view of a Λ = 22 and Λ = 88 nm reacted foil, respectively. The increased grain size for the Λ = 88 nm sample (**c**) is remarkable. The substantially decreased thickness of the oxide and the Ru interlayer is also notable in said multilayer. (**b**) Selected area diffraction (SAD) pattern of a volume in (**a**) including the equiaxed microstructure of the foil interior as well as the double-structured surface scale. (**d**) SAD analysis of the bulk of the foil in (**b**) reveals a single-phase RuAl microstructure.

After identifying the reaction as a one-step transformation, we need to locally cross-check the microstructure as being single-phased which was defined as a second ductility indicator. Figure 6(a) presents a representative cross-sectional view of the sample with Λ = 22 nm. It reveals an equiaxial microstructure and no evidence of the original multilayer structure. According to Trenkle *et al.* this indicates temporary liquid phase presence during the reaction^27^. Hence, the observed as-reacted microstructure also suggests temporary melting during the reaction which corroborates the transformation mechanism developed above. Closer analysis of Fig. 6(a) reveals a nanosclaed double-structured surface scale on top of the equiaxial microstructure. The surface scale comprises an interlayer of dark contrast covered by a surface layer of bright contrast. To identify the individual phases we performed selected area diffraction (SAD) within a volume including the equiaxed microstructure as well as the double-structured surface scale. The result is shown in Fig. 6(b) and confirmes the presence of B2-RuAl, Ru, and Al_2_O_3_. In the Supplementary 2 we present a detailed analysis and identify the two phases of the surface scale to be Ru and Al_2_O_3_ and the bulk of the foil as RuAl.

The doubled structured surface scale is characteristic for the oxidation of RuAl. Surface oxidation is generally observed in RE/TM and Ni/Al reactive foils when reacted in air. Mc Donald *et al.* demonstrate that oxidation follows the self-propagating reaction^12^. Whereas the X-ray diffraction experiment of the present study does not detect any oxidation process during the initial RuAl formation in the first 5 ms, the temperature measurements, which are discussed in Supplementary 1, indicate reactions with the atmosphere in later stages >5 ms. It is therefore suggested that the observed surface oxidation occurs in later reaction stages after the initial RuAl formation. Current research explores ways to tailor the degree of surface oxidation because it is of interest for various applications. Whereas latest studies demonstrated that chemistry or stoichiometry variation affects oxidation^40^,^41^, we will now show that Λ variations (while keeping chemistry constant) can also be utilized to control oxidation. Intriguingly when increasing Λ from 22 nm to 88 nm the double-structured oxidation scale decreases to a negligible amount, Fig. 6(c). In addition to the decrease of the surface scale thickness, the RuAl grain size substantially increases. We associate the reduction of the oxidation layer with slower oxidation kinetics due to the increased RuAl grain size (details see Supplementary 2).

Despite the presence of a nanoscaled oxidation scale we highlight the single-phase as-reacted RuAl microstructure in the bulk of the foil. This was earlier defined as one indicator for ductility. The grain size of the RuAl grains increases with the bilayer thickness (compare Fig. 6(b,d)). A possible explanation for the enhancement of the grain size is the considerable reduction in Ru/Al interface density in large-Λ samples. As pointed out above, RuAl formation starts with nucleation at the Ru/Al interface. Consequently, a decrease in Ru/Al interface density decreases the nucleation site density enabling the formed grains to grow to a larger extend up to impingement (assuming that the temperature does not significantly vary). In this context, it is important to note that the foils transform in one step without the formation of any secondary transient intermetallic (as shown above). This pathway enables us to directly affect the nucleation site density via the initial multilayer period and opens up the way to a systematic grain size control of the reacted foil. Interesting from a fundamental point of view is the observation of rectangular shaped pores within the grains of both microstructures. The edges of the pores are well parallel aligned within the grain and faceted towards the (001), (210) and (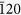) planes of the surrounding RuAl matrix. During rapid cooling thermal vacancies are quenched in^42^. Subsequent self-assembling mechanisms result in pore formation^43^ (for detailed discussion see Supplementary 3).

## Conclusion

In conclusion, the equiatomic Ru/Al system shows the best integration between maximal RT ductility, off-stoichiometry acceptance and maximal energy density. In finding evidence for the hypothesis, we report about self-propagating reactions in equiatomic freestanding Ru/Al multilayers and their fundamental characterization. The bilayer thickness enables tailoring propagation velocity and reaction temperature. The final grain size of RuAl decreases with the initial multilayer period. We found a bilayer insensitive direct formation of RuAl that provides the essential prerequisite for ductile foils. As a consequence, Ru/Al multilayers serve as an optimal candidate for improved joining and applications in MEMS. Finally, this study introduces the utilization of a family of Ru/Al-based systems to tailor the properties to suit specific requirements. As an example, the precise tuning of Ru/Al multilayers to modulate energy density, reaction temperature, ductility and final microstructure offers the implementation of a new benchmark in the widespread use of reactive multilayers as a local heat source in industrial applications.

## Methods

### Sample preparation and reaction conditions

Ru/Al multilayers with nominal bilayer thicknesses of 22, 44, 66, 88 and 178 nm were deposited on Mica substrates by DC magnetron sputtering using elemental high-purity Ru and Al targets in a von Ardenne PPS-A 200 system. The total thickness of all samples was chosen to be 6 μm. The base pressure was 10^−6^ mbar. The Ar gas sputtering pressure was 3 × 10^−3^ mbar. To construct the multilayers, the substrate was alternately rotated under the corresponding target. The Ru sputtering rate was 0.2 nm/s, the Al sputtering rate was 0.24 nm/s. Afterwards, the samples were peeled off the substrate. The experiments were done with freestanding specimens. The samples used had a size of approximately 3 mm × 2 cm. All experiments were performed with samples of the same charge to avoid fabrication inhomogeneities.

Experiments were done in air or in a custom-built vacuum chamber under a pressure of 10^−5^ mbar. In air, the reactions were locally ignited with an electric spark whereas those in vacuum are started with a high-energy Nd:YAG nanosecond pulse laser working at the third harmonic with a wavelength of 355 nm (Spectra Physics Quanta Ray Pro 290).

### Measurement of reaction front velocity

To measure the reaction velocity in plan view, successive images were taken with the high-speed camera MotionScope M3 - IDT, Inc. with a time resolution of 92 μs. For every bilayer thickness the reactions of at least four specimens were measured. Since the reaction front exhibits a time-invariant curvature (at least for the observed duration), we calculate the velocity by sizing the travelling distance of the middle position of the depicted reaction front between the individual frames. We further verified that the position had the same grey scale value in every frame. Finally, the reaction velocities were averaged for every Λ group.

### *In situ* time-resolved X-ray diffraction

The experiments were done in air at the Materials Science Beamline (X04SA) of the Swiss Light Source at Paul Scherrer Institue in Villigen, Switzerland^44^. The X-ray beam of a photon energy of 12.4 keV was focused at the sample on an area of 1 mm × 0.5 mm. The reaction was initiated at a position between 5–10 mm away from the focus of the X-ray beam. The diffractograms were recorded in transmission. Important for the measurements was the combination of high brilliant synchrotron radiation with the high-speed MYTHEN II micro strip detector^31^. The latter uniquely allows the successive measurement of ten large 2θ-range diffractograms (±60°) with a high angular (0.004°) and sub-μs temporal resolution. The read-out time of the individual diffractograms is 250 μs. For statistical reasons the temporal resolution was chosen to be 250 μs, i.e. the individual diffractograms were recorded over 250 μs. It is important to note that the chosen detector setup allowed the measurement without any dead time despite the mentioned read-out time. For that purpose the symmetric diffraction geometry was exploited by dividing the middle-positioned detector into “upper” and “lower” halves. Using 250 μs time-shifted measurements of both halves, the phase evolution was monitored continuously.

### High-speed Pyrometry

The temperatures of the reacting multilayers were measured in plan view with the high-speed digital pyrometer Metis HS09 (Sensortherm GmbH). The spectral range is 0.7–1.1 μm. The temporal resolution is 70 μs. The spot size has a diameter of 1 mm. As the emissivity during the first microsecond of the reaction varies strongly, it was set to a value of 1 during the experiments. Consequently, the measured data is minimal and underestimates the real temperatures.

### Microstructural Analysis

Cross-sections of the reacted samples for transmission electron microscopy (TEM) studies were prepared using a FEI dual beam workstation (Helios Nanolab). Scanning TEM was used to image the microstructure. In addition selected area diffraction (SAD) in the TEM (JEOL 2010F) with 200 kV acceleration voltage was used to provide proof of the single-phase RuAl microstructure and the faceting of the mesopores.

### Computer Simulations

Computer simulation of a self-propagating reaction was done with molecular dynamics method implemented in the LAMMPS^45^ code. The embedded-atom method^46^(EAM) potential was used to compute interatomic interactions. The Al-Al potential term was described by Voter and Chen^47^, for Ru-Ru and Al-Ru terms from the modified potential by J. A. Rifkin^48^ were used. The new fitting of the original parameters from this work was done for better reproduction of the elastic constants, melting temperature and the enthalpy of mixing for these compounds. The simulation box comprised two layers of Al (FCC lattice) and Ru (HCP lattice) with dimensions, 4 nm × 83 nm × 22 nm in *x*, *y* and *z* directions, correspondingly. The interface between the two metals was perpendicular to the *z*-axis, the thickness of the layers was chosen to provide the equal amount of the Ru and Al atoms in the sample. The periodic boundary conditions were applied in the *x*- and *z*-directions. The sample initially was relaxed at the temperature *T* = 300 K and the pressure *p* = 0 bars and then the excitation was induced at one of the ends of the samples (in *y*-direction) by rapid heating – up to 6000 K during 0.12 ns – to ignite the reaction. Then the system was simulated with constant volume and energy conditions (*NVE* ensemble).

The lattice type was defined by performing the common neighbor analysis (CNA)^49^ with adaptive cutoff radius for the nearest neighbors’ determination^50^. RuAl B2 phase was defined as BCC lattice with 8 atoms of one type on the first coordination shell and 6 atoms of other type on the second coordination shell.

## Additional Information

**How to cite this article**: Woll, K. *et al.* Ru/Al Multilayers Integrate Maximum Energy Density and Ductility for Reactive Materials. *Sci. Rep.* **6**, 19535; doi: 10.1038/srep19535 (2016).

## Supplementary Material

Supplementary Information

Supplementary video

## Figures and Tables

**Figure 1:**
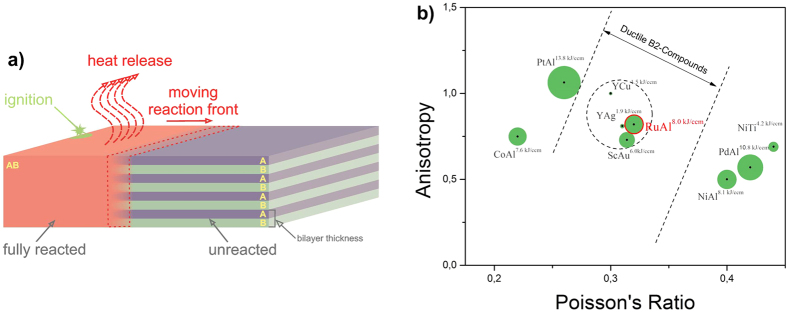
Ru/Al reactive multilayers serve as a model material to integrate both maximum energy density and ductility. (**a**) Principle schematic of reactive foils. The self-propagating reaction (A + B → AB) is locally ignited at the left side and moves under heat release to the right. (**b**) Ashby map to select a B2 compound-forming material system with an optimal combination of maximal stored energy and compound ductility. The anisotropy A^−0.5^, where A denotes the anisotropy factor of the compound, is plotted against its Poisson’s ratio. The circular dotted line is a guide to the eye and denotes the region where the recently discovered ductile intermetallics cluster. The size of the bubble for every compound is proportional to its maximal stored energy. The Ru/Al system shows a substantially improved trade-off between stored energy and compound ductility compared with studied systems thus far.
