# Supplementary material for: Ru/Al Multilayers Integrate Maximum Energy Density and Ductility for Reactive Materials
Source: Sci Rep. 2016 Jan 29;6:19535. doi: 10.1038/srep19535 (PMC4731779; doi:10.1038/srep19535)
Supplement: Supplementary Information [file srep19535-s1.pdf]

Ru/Al Multilayers Integrate Maximum Energy Density and Ductility for Reactive Materials

K. Woll[1), #,*], A. Bergamaschi[2)], K. Avchachov[3)], F. Djurabekova[3)], S. Gier[4)], C. Pauly[1)], P. Leibenguth[1)], C.Wagner[4)], K. Nordlund[3)], F. Mücklich[1),]

[1)] Functional Materials, Department of Materials Science, Saarland University, 66123 Saarbrücken, Germany
[2)] Paul Scherrer Institute, 5232 Villigen-PSI, Switzerland
[3)] Materials Physics, Department of Physics, University of Helsinki, 00014 Helsinki, Finnland
[4)] Experimental Physics, Saarland University, 66123 Saarbrücken, Germany

Supplementary Material

1. Discussion of the temperature profiles

Representative temperature evolutions during the reaction as a function of Λ are presented in Figure 3. The temperature drop for the small Λ multilayer is attributed to the loss of chemical energy due to fabrication-induced interface interdiffusion. Interdiffusion reduces the heat of reaction (heat of formation of RuAl) by the heat of mixing of the elements. Consequently, the maximal achievable reaction temperature is reduced. This effect becomes more prominent with smaller Λ. During cooling two characteristic slope variations are visible. First, for samples with $\Lambda \geq 44$ nm, a cooling rate delay occurs at ≈ 1,830 °C. This indicates a phase transformation. The composition is slightly Al-rich. According to the Ru/Al equilibrium diagram the microstructure at such high temperatures is a mix of mainly solid RuAl and a small amount of Al-rich liquid. Consequently, the delay denotes the solidification of the Al-rich liquid. In the case of the $\Lambda = 22$ nm specimen, this delay is not present. Due to the loss of chemical energy the temperatures decrease below the solidus temperature. As a result, the product remains in its solid state during the reaction. For samples with $\Lambda \leq 44$ nm another delay in the cooling rate occurs at ≈ 1,640 °C. Temperature measurements in vacuum, however, do not show said delay (see Figure S1).

[#] now at: Karlsruhe Institute of Technology (KIT), Institute of Applied Materials (IAM), 76344 Eggenstein-Leopoldshafen, Germany
[*] corresponding author: Karsten.woll@kit.edu

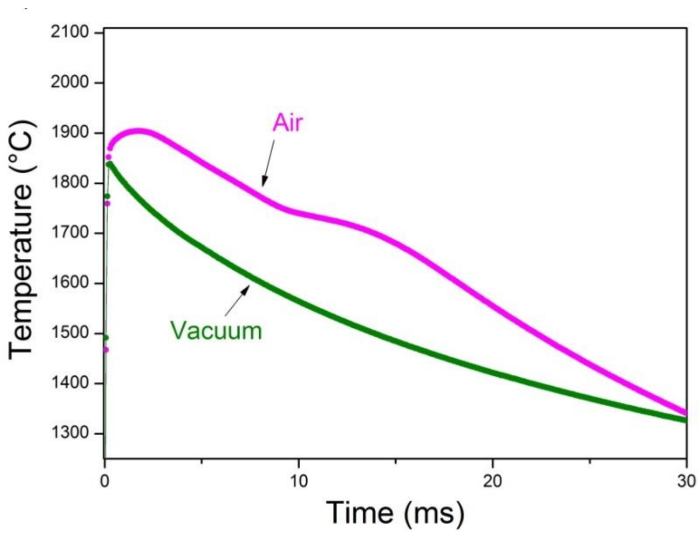

Figure S1: High-speed temperature measurements. Comparison between the temperature profile of a Λ = 22 nm multilayer reacted in air and vacuum. Note the absence of delay at about 1600 °C when reacted in vacuum.

Thus, an interaction with the surrounding air is suggested. Indeed the microstructural analysis of this sample proves a higher degree of oxidation at the surface compared to samples with Λ ≥ 88 nm. Instead no oxidation is observed in the vacuum reacted multilayer (for details see Supplementary 2).

2. Interaction with the surrounding atmosphere of reacting multilayers

Interaction with air of reacting Ru/Al multilayers and surface oxidation was observed in multilayers with bilayer thicknesses Λ ≤ 44 nm. At the surface, an Al_2O_3 layer of 120 – 180 nm thickness is visible. Adjacent to the oxide layer, an Ru interlayer is observable.

First, Figure S1 a – d reveals the formation of Al_2O_3 via TEM bright/dark field imaging. The bright field image using spot #1 is shown. Figure S1 b) depicts the selected area diffraction pattern. Spots #1 and #2 which were identified as α-Al_2O_3 reflexions were used for dark field imaging (Figure S1 c) and d)). Figure S1 c) and d) proves that the marked area of the surface layer consists of α-Al_2O_3. The formation of these phases proves the oxidation of RuAl when reacted in air. Second, Figure S2 shows, for cross-checking purposes, the microstructure of a Λ = 22 nm sample reacted in vacuum. No surface layer is observed. Hence, it can be

unambiguously concluded that the observed oxide layer in air-reacted samples occurs as a result of interaction with the surrounding atmosphere.

Oxidation studies on bulk material demonstrate the similar layered structure [15]. Outward Al diffusion to the oxidizing surface results in Al depletion of RuAl in near-surface regions where its composition is shifted to Ru-rich values. Once the solubility limit is reached, elemental Ru precipitates (according to the equilibrium diagram).

The Al supply which is needed for the oxide to grow occurs via volume and grain boundary diffusion. The latter is increased about 3 orders of magnitude and is more dominant with decreasing grain size. This accelerates the oxidation kinetics for smaller grain sizes and a thicker oxide scale is formed. As a significant larger grain size is observed in reacted Λ = 88 nm multilayers it is suggested that the grain boundary diffusion is less prominent in these samples. Hence, Al diffusion occurs mainly via the grain volume which decelerates the Al diffusion compared to the Λ = 22 nm sample. This results in a significant thinner oxide scale.

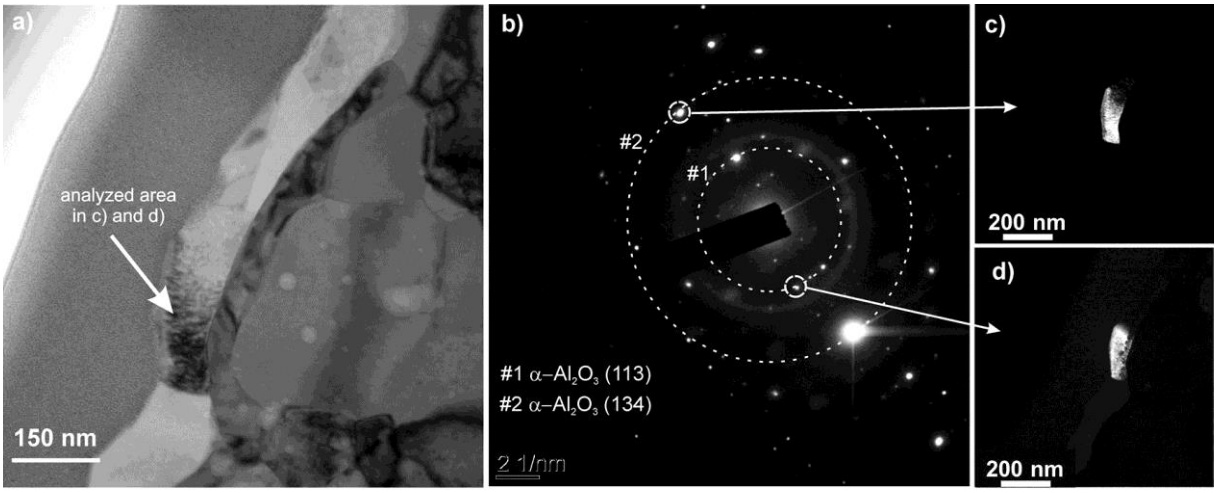

Figure S2: Surface layer analysis of a reacted specimen with Λ = 22 nm. a, Bright field image that shows the studied region of the sample. The marked area is analyzed via dark field imaging (Figure S1 c) and d)). **b,** SAD pattern of the surface near area in **a,**. Spots #1 and #2 denote α-Al_2O_3 reflections. **c,** and **d,** dark field images using spots #1 and #2.

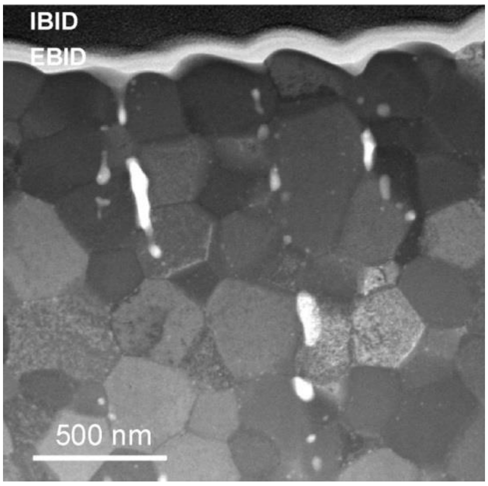

Figure S3: Microstructure of vacuum reacted Ru/Al multilayer. Cross-sectional microstructural STEM image of a vacuum reacted specimen with Λ = 22 nm. EBID (electron beam induced deposition) and IBID (ion beam induced deposition) denote protective Pt-cap layers needed for TEM foil preparation. The absence of an oxidation layer on the surface is important.

3. Mesopore formation during self-propagating reactions

Interesting from a fundamental point of view is the observation of rectangular-shaped pores within the grains of both microstructures. Figure S3 a) focuses on one grain of the Λ = 88 nm sample. The edges of the pores are well parallel aligned within the grain. Selected area diffraction reveals that the voids are faceted towards the (001), (210) and ($\bar{1}$20) planes of the surrounding RuAl matrix (Figure S3b)). Faceted voids were also observed in other intermetallic aluminides, like NiAl [16], FeAl [17] and CoAl [18], after annealing of rapidly quenched microstructures. During rapid cooling, from high temperatures, a large amount of thermal vacancies, with concentrations up to several percent at temperatures near the melting point, are quenched in. Subsequent annealing results in clustering and faceted pore formation via self-assembling mechanisms. It is also reported that faceted voids are already present after quenching (without subsequent annealing) [16]. For this case, pore formation along sub-cell boundaries is suggested. The vacancy concentration of stoichiometric RuAl bulk samples quenched from 1200 °C was experimentally determined at 2.5 % [19]. As the temperatures in the

present samples are substantially higher it is anticipated that the vacancy concentrations are > 2.5 %. According to Zakaria et al. faceted void formation is already possible at lower concentrations of quenched-in vacancies (0.49 % at 1300 °C) [16]. Consequently, it is deduced that rapid cooling of the RuAl microstructure also results in faceted void formation along the mentioned crystallographic planes. Taking into account that the B2 structure consists of two interpenetrating cubic primitive lattices, it should be noted that the (001), (210) and $(\bar{1}20)$ planes are occupied by only one element (Al or Ru) with maximized dense package (generally dense packed planes are energetically favoured). Therefore presumably, there exists a site preference for vacancy agglomeration towards one of the sublattices. It was experimentally shown for Al-rich RuAl bulk samples that vacancies exist exclusively on the Ru-sublattice [20]. This is also corroborated by first principle studies on the point defect formation enthalpy [21.] Accordingly, the latter is more than a factor 3 lower for vacancies in the Ru-sublattice compared to that of the Al-sublattice. Hence, it can be concluded that the quenched-in vacancies in the present specimen condense mainly on the Ru sublattice. The formation of Al-occupied-surfaces in the RuAl matrix appears to be energetically favoured.

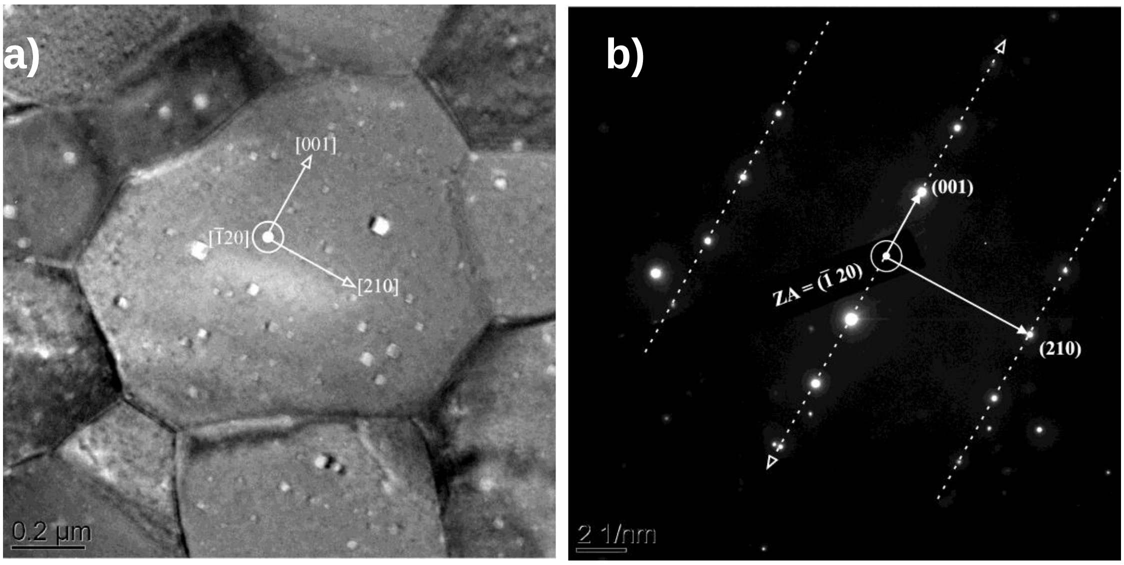

Figure S4: Pore analysis in terms of faceting. a, Cross sectional TEM image of a sample with Λ = 88 nm. The grain contains parallel aligned rectangular pores. **b,** $(\bar{1}20)$ zone axis pattern of the grain shown in **a,**. The main reflexions (001) and (210) are parallel aligned to the boundary planes of the pores in **a,**.

4. Video showing the initial states of intermixing after ignition

The Supplementary Material contains a video showing the results of the molecular dynamics simulations. The latter simulate the early stages (first two nanoseconds) of intermixing between Al and Ru after ignition at the left hand side.

References

[1] M. Zakira und P. Munroe, *J. Mater. Sci. 2002*, 71

[2] H. Gobran, K. Liu, D. Heger und F. Mücklich, *Scr. Mater. 2003,* 49, 1097.

[3] H. Gobran, Synthesis and Characterization of single-phase B2 structured RuAl intermetallic compound by powder metallurgy (Issue 4), F. Mücklich, W. Arnold, R. Busch, R. Clasen, S. Diebels, M. Kröning, W. Possart, H. Schmidt and H. Vehoff, Eds., Aachen: Shaker Verlag, 2006.

[4] S. Prins, R. Arroyave und Z.-K. Liu, *Acta Mater. 2007,* 55, 4781.

[5] R. Nakamura, K. Yoshimi und S. Tsurekawa, *Mater. Sci. Eng. A, 2007,* 1036

[6] M. Tsunekane, K. Yoshimi und K. Maruyama, *Acta Mater. 2008,* 56, 3162.

[7] F. Soldera, N. Ilic, S. Brännström, I. Barrientos, H. Gobran und F. Muecklich, *Oxid. Met. 2003,* 59, 529.

[8] J. Trenkle, L. Koerner, M. Tate, N. Walker, S. Grunder, T. Weihs und T. Hufnagel, *J. Appl. Phys. 2010,* 107, 113511(1).

[9] J. McDonald, M. Rodriguez, E. Jones und D. Adams, *J. Mater. Res. 2010,* 25, 718.

[10] F. Mücklich, N. Ilic und K. Woll, *Intermetallics 2008,* 16, 593.
